# Age of First Menstruation

**Published:** 1902-03

**Authors:** 


### Age of First Menstruation.

Dr. Geo. J. Engelmann, *N. Y. Medical Journal,* has prepared an extremely interesting paper on this subject. He finds that American women as to the time of functional development are more precocious than are the women of other continents in the same zone, and that they are more precocious than the people from whom they are descended. On this continent the average age is 14, while in Europe it is 15.5. Climate does not influence the time of pubertal development in the temperate regions of this continent. Here, racial characteristics as to the time of development rapidly fade away. Girls of the working classes develop sooner than do girls of the wealthy classes. In this country mentality, surroundings, education and nerve stimulation are the factors that determine the time of development. R.

